# Retraction: Arsenic in the Food Chain in Pakistan: Assessing Risks to Human Health and Ensuring Food Security Through Comprehensive Contamination Mitigation Strategies

**DOI:** 10.7759/cureus.r207

**Published:** 2025-11-25

**Authors:** Syed Shameel Ahmed Quadri, Rabia Ali, Anderson Mesidor, Imam Ali Shah, Nimra Raqeeb, Muhammad Omer Afzal, Muhammad Rizwan, Tariq Rafique, Saba Nosheen

**Affiliations:** 1 Political Science, University of Karachi, Karachi, PAK; 2 General Practice, SHED Foundation Hospital, Karachi, PAK; 3 Medical Education, Université d'État d'Haïti, Port-au-Prince, HTI; 4 Internal Medicine, Chandka Medical College, Shaheed Mohtarma Benazir Bhutto Medical University, Larkana, PAK; 5 Zoology, Mirpur University of Science and Technology, Mirpur, PAK; 6 Center for Agricultural Biochemistry and Biotechnology, University of Agriculture Faisalabad, Faisalabad, PAK; 7 School of Energy Science and Engineering, Central South University, Changsha, CHN; 8 Medical Affairs, Dadabhoy Institute of Higher Education, Karachi, PAK; 9 Editorial Board, Research Journal of Innovative Ideas and Thoughts, Faisalabad, PAK

The Editors-in-Chief have retracted this article. An investigation by the journal found evidence of authorship manipulation. The Editors-in-Chief therefore no longer have confidence in the provenance and reliability of the article contents.

